# Unsupervised speech recognition through spike-timing-dependent plasticity in a convolutional spiking neural network

**DOI:** 10.1371/journal.pone.0204596

**Published:** 2018-11-29

**Authors:** Meng Dong, Xuhui Huang, Bo Xu

**Affiliations:** 1 School of Automation, Harbin University of Science and Technology, Harbin, Heilongjiang, China; 2 Research Center for Brain-inspired Intelligence, Institute of Automation, Chinese Academy of Sciences, Beijing, China; 3 School of Artificial Intelligence, University of Chinese Academy of Sciences, Beijing, China; 4 Center for Excellence in Brain Science and Intelligence Technology, Chinese Academy of Sciences, Beijing, China; Hong Kong Baptist University, HONG KONG

## Abstract

Speech recognition (SR) has been improved significantly by artificial neural networks (ANNs), but ANNs have the drawbacks of biologically implausibility and excessive power consumption because of the nonlocal transfer of real-valued errors and weights. While spiking neural networks (SNNs) have the potential to solve these drawbacks of ANNs due to their efficient spike communication and their natural way to utilize kinds of synaptic plasticity rules found in brain for weight modification. However, existing SNN models for SR either had bad performance, or were trained in biologically implausible ways. In this paper, we present a biologically inspired convolutional SNN model for SR. The network adopts the time-to-first-spike coding scheme for fast and efficient information processing. A biological learning rule, spike-timing-dependent plasticity (STDP), is used to adjust the synaptic weights of convolutional neurons to form receptive fields in an unsupervised way. In the convolutional structure, the strategy of local weight sharing is introduced and could lead to better feature extraction of speech signals than global weight sharing. We first evaluated the SNN model with a linear support vector machine (SVM) on the TIDIGITS dataset and it got the performance of 97.5%, comparable to the best results of ANNs. Deep analysis on network outputs showed that, not only are the output data more linearly separable, but they also have fewer dimensions and become sparse. To further confirm the validity of our model, we trained it on a more difficult recognition task based on the TIMIT dataset, and it got a high performance of 93.8%. Moreover, a linear spike-based classifier—tempotron—can also achieve high accuracies very close to that of SVM on both the two tasks. These demonstrate that an STDP-based convolutional SNN model equipped with local weight sharing and temporal coding is capable of solving the SR task accurately and efficiently.

## Introduction

Automatic speech recognition is the ability for a machine to recognize and translate spoken language into text. It is a challenging task since the speech signal is high variable due to different speaker characteristics, varying speaking speed, and background noise. In recent years, artificial neural networks (ANNs), especially deep neural networks, have outperformed traditional Gaussian mixture models and became the predominant method in speech recognition area [1].

ANNs are inspired by features found in brain. They consist of multiple layers of artificial neurons which are able to learn data representations from the input data by gradient descent algorithms [2, 3]. In some scenarios, ANNs can reach or surpass human level performance. Despite the biological inspiration and high performance, ANN models are fundamentally different from what are actually observed in biology in two main aspects. Firstly, the artificial neurons in ANNs communicate with each other by sending real numbers which can be seen as their firing rates. In contrast, neurons in biological neural networks communicate via spikes or pulses. Secondly, the standard training method for ANNs is backpropagation [4], which update weights of neurons calculated from non-local error signals and weights of downstream synapses. However, it seems quite implausible that this process of non-local information propagation would occur in the cortex [5], in which neurons just communicate with each other based on spikes from direct connections, and the synaptic strengths are generally modified by activities of corresponding pre- and post-synaptic neurons, e.g. spike-timing-dependent plasticity (STDP) [6–10]. STDP was found in experiments in many cortex regions [6, 9, 10] and was believed to be a basic principle for the formation of recognition and memory in human brain. Besides, compared to the brain’s energy efficiency, both training and execution of large-scale ANNs need massive amounts of computational power to perform single tasks.

For these reasons, there has been a growing interest in spiking neural networks (SNNs) recently. Like in the brain, a neuron in the SNNs fires only when its membrane potential reaches its threshold. When a neuron fires, its post-synaptic neurons receive the spike and update their potentials. When implemented on neuromorphic platforms like TrueNorth [11], the SNNs can operate with ultra-low power consumption. Although in both theoretic [12] and model studies [13–17], SNNs have been shown their powerful ability and advantages in kinds of machine learning tasks, the development on SNN models is still in a primary stage compared with ANNs.

For the speech recognition task, several SNN models have been proposed, which have either recurrent connections or feedforward connections. For the recurrent SNN models, a popular approach is the liquid state machine (LSM) [18–25], which is one of two types of reservoir computing [26]. A typical LSM consists of three layers (input layer, reservoir layer, and readout layer). The reservoir layer is a collection of recurrently and randomly connected spiking neurons, whose connections can be learned by synaptic plasticity rules [23–25]. The reservoir can serve as a function of short-term memory for storing temporal input information in a higher dimension, this makes LSMs suitable for the speech recognition task [19–22]. Nonetheless, in these models, the feature extraction step is very obscure due to the random projection and there is no concept of receptive field when comparing to the sensory system. Moreover, LSMs increase the separability of data by mapping them into a higher dimension, which is not very efficient. For the class of SNN models with feedforward connections, Wade et al. [27] presented a synaptic weight association training (SWAT) algorithm for SNNs, which merges the Bienenstock–Cooper–Munro (BCM) learning rule with STDP. But an additional training neuron is used to train the synaptic weights of output neurons and removed after training, which is not biologically reasonable. Tavanaei and Maida [28] proposed a two layer SNN model which learns to convert a speech signal into a distinguishable spike train signature. Their model is trained by switching between Hebbian and anti-Hebbian STDP rule based on the label of current sample. The performance of this model was not good, and their encoding method is inefficient. Another SNN model proposed by Tavanaei and Maida [29] uses probabilistic STDP to extract discriminative features from speech signals. Their model achieved high performance on the speech recognition task, but the convolutional layer which extracts primary auditory features uses hand-crafted Difference-of-Gaussian (DoG) filters, which is unlikely to happen in biological auditory systems.

Therefore, for the purpose of both biological plausibility and energy efficiency, here we proposed a feedforward SNN with STDP and fast temporal encoding scheme for the speech recognition task. Our model was inspired by [30] and [31], it consists of a convolutional layer and a pooling layer. In the convolutional layer, the receptive fields of neurons are learned by STDP to extract acoustic features from speech signals. Moreover, the weights in the convolutional layer are shared locally to better capture the features of spoken words. The pooling layer performs a pooling operation to reduce the size of feature maps in the convolutional layer. For a fast and efficient encoding, the time-to-first-spike coding scheme [32] is adopted in our model. Finally, the output of the pooling layer is used to train a linear classifier. We evaluated our model with a linear classifier on the isolated spoken word recognition task on the TIDIGITS dataset [33], and achieved an accuracy which outperformed all other SNNs and was comparable to the best result of ANNs. More analysis on network outputs reveal that STDP is able to extract features of speech signals to make speech data more separable. Furthermore, the validity of our model can also be well maintained by changing the classifier to tempotron [34] and the dataset to the TIMIT dataset [35].

## Methods

**Fig 1:**
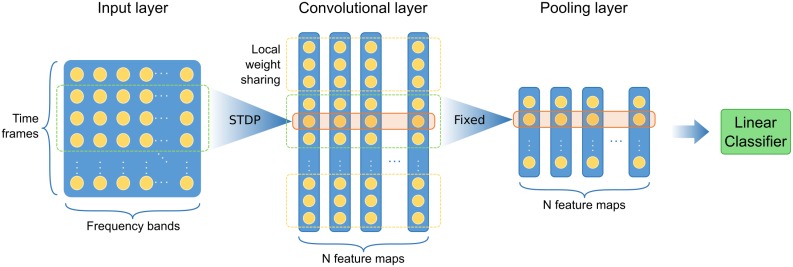
Architecture of proposed spiking neural network (SNN). The network consists of an input layer, a convolutional layer, and a pooling layer. The input layer converts the Mel-Frequency Spectral Coefficients (MFSC) of speech signal into spikes using the time-to-first-spike coding scheme. The convolutional layer contains multi features maps which are responsible for detecting different features, and their input weights are learned by spike-timing-dependent plasticity (STDP). Each feature map in the convolutional layer is divided into non-overlapping sections which have shared input weights. The pooling layer compresses the output of the convolutional layer, and its output is classified by a linear classifier.

Our network model consists of three layers, which is illustrated in the architecture diagram Fig 1. The input layer converts the speech signal into spikes using the time-to-first-spike coding scheme, the convolutional layer learns acoustic features from the input by STDP learning rule, and the pooling layer compresses the information while providing the translation-invariance. The details of each layer will be explained in the following sections.

### Input encoding

For the SNN to process, analog speech signal is needed to be encoded into discrete spikes. First we extract features from the raw signal to get a better representation, which is the first stage of almost any speech recognition system.

As the encoder in human auditory system, the cochlea receives sound in the form of vibrations. When the sound vibration is transmitted to the basilar membrane, different points along the membrane resonate to specific frequencies. As a result, the hair cells protruded from the basilar membrane also have a tonotopic sensitivity, which can be modeled by an array of band-pass filters known as filter banks. The filter-bank-based Mel-Frequency Cepstral Coefficients (MFCCs) [36] is the most common feature extraction method in machine learning area, briefly speaking, it is a discrete cosine transform (DCT) of a log power spectrum on a nonlinear mel-scale of frequency. The target of the DCT step is to get the envelope of spectrum but it destroys the locality of features. However, the convolutional layer in our network (as well as the convolutional layer in a traditional convolutional neural network) needs the locality of features to work properly. So instead of MFCCs, we choose Mel-Frequency Spectral Coefficients (MFSCs), which omit the DCT step in the extraction of MFCCs and preserve the locality. Specifically, we compute the Mel-scaled filter banks by applying triangular filters on a Mel-scale to the power spectrum, and take the logarithm of the result. Another issue during the generation of features is that the audio signals have variable length, but the input layer of the model only contains a fixed number of neurons, so we use different window length in the Fourier transform step during the MFSC feature extraction to get an input of fixed length. Hence, all data samples with different temporal lengths are converted into MFSCs with same lengths. This time-warp-invariant information processing is the basic ability of auditory systems [37].

The sensitivity of a single auditory neuron depends not only on frequency, but also on time, as described by the spectro-temporal receptive field (STRF) [38, 39], which means the neurons sensitive to the same frequency band may have different response latencies. To simulate this behavior, we organize the neurons in the input layer into a two-dimensional *M* × *N* array, where each row is a time frame and each column a frequency band. The dynamics of neuron (*m*, *n*) depends on the time frame *m* and the frequency band *n*.

**Fig 2:**
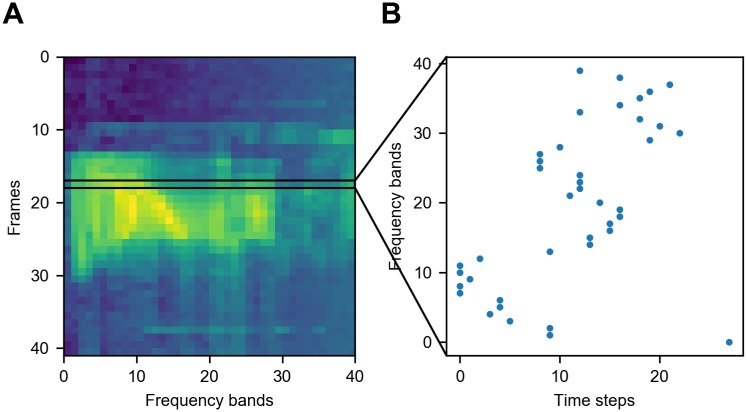
The input coding of the SNN. (A) The MFSC spectrogram of the spoken digit “one”. The horizontal axis represents the index of frequency bands, and the vertical axis represents the time frames. (B) The spike coding of one frame (the row of pixels inside the black box) in Fig A. The MFSC features are encoded by the time-to-first-spike coding scheme. The higher the feature value, the earlier the neuron fires. Note that the time axis of Fig B is irrelevant to time axis of Fig A.

For each neuron in the input layer, they must convert the analog input signal into discrete spikes. Various neural encoding schemes can be used for this purpose. The most popular method is to convert the input into a Poisson-distributed spike train, which encodes the information in the neuron firing rates, while all the information possibly contained in the temporal structure of the spike train is neglected [14, 40]. This method can be time-consuming and energy-inefficient. Instead, we use the time-to-first-spike coding scheme, as shown in Fig 2, in this model information is encoded in the response latency of the time of first spike relative to the stimulus onset. Such coding scheme has been discovered in several sensory systems in the brain, including the auditory [41, 42], visual [43, 44], and somatosensory [45–48] systems. In this strategy, each neuron only needs to emit a single spike to transmit information during a presentation of an input sample, while all following spikes can be ignored or be assumed as inhibited by an inhibitory feedback connection. For simplicity, we just shut off the neuron as soon as it fires a spike. Each input neuron (*m*, *n*) converts the intensity of the frequency band *n* in the time frame *m* into the time of the first spike. The higher the intensity, the earlier the neuron fires. With this temporal encoding scheme, our SNN is capable of high-speed and low-energy data processing compared to rate coding, since a post-synaptic neuron can encode the whole information from a pre-synaptic neuron as soon as the former receives the first spike from the latter, while the rate encoding can not estimate the information in a very short interval due to the non-instantaneity of mean firing rate.

### Convolutional layer

The spiking neuron model used in the convolutional layer is the integrate-and-fire (IF) model, which is simple yet captures some fundamental characteristics of real neurons. The IF model assumes that a neuron integrates input spikes from presynaptic neurons and increase its membrane potential each time a spike arrives. When the membrane potential exceeds a specific threshold *V*_*thresh*_, the neuron fires a new spike, and the membrane potential is reset to its rest level *V*_*rest*_. The update of the membrane potential of an IF neuron can be described as [30, 31]:

$$\begin{array}{c} V\left(t\right)=V\left(t-1\right)+W^{T}\cdot S\left(t-1\right) \end{array}$$ (1)

where *V* is a vector of all neurons’ membrane potential, *W* is the input synaptic weight matrix, and *S* is a vector represents the spikes in the last time step. After each sample is processed, we reset the membrane potential *V* to *V*_*rest*_, *V*_*rest*_ = 0.

The concept of convolution here involves two main properties: local connection and weight sharing, which is similar to the artificial convolutional neural networks. Each neuron in the convolutional layer connects to a region of previous layer which spans the entire frequency range but only covers a small period of time. The convolutional layer consists of several sub-layers which we will refer as feature maps, see Fig 1. The neurons on the same location of different feature maps share the same input window, only with different presynaptic weights. Another property of the convolutional layer is weight sharing, which usually means the same weights are used by many neurons in the same feature map. Thus computation of neuronal potentials can be viewed as a convolution of input signal with the shared weights. Weight sharing enables the convolutional layer to learn and recognize features of the input data regardless of their absolute position within the input. Different feature maps are responsible for detecting different features determined by their presynaptic weights. With these properties, the parameters to be trained are reduced and the training becomes more efficient.

### Local weight sharing

The weight sharing scheme described above is usually used in a global manner, which means that all neurons in a feature map share the same weights. This is the standard way in the image processing task, since empirically the same feature can appear at any location of the image. However, the features appearing at different time periods of the speech signal have a characteristic of locality. For example in English, the prefixes and suffixes are at different positions in a word and have different acoustic features. Chinese characters almost always begin with a consonant and end with a vowel, which also have different features. Therefore, a better strategy may be to share weights only among nearby convolutional neurons, but use separate sets of weights for different time periods of a spoken word, respectively. The local shared weights in the temporal domain were used for calculating the inputs of the corresponding convolutional neurons, and this strategy is called as local weight sharing. Fig 1 shows the local weight sharing strategy used by our model. The convolutional layer is divided into several non-overlapping sections, and each section receives input from different overlapping time periods with their own shared weights, but still spans all feature maps. As a result, the local shared weights should be able to learn local features from the corresponding time period. The output of convolutional neurons in each section are then pooled together, indicating the existence of the learned feature of this section, which will be discussed later.

### Learning weights with STDP

The weights of convolutional layer are initialized by drawing from a Gaussian distribution. When the network is in the training process, the weights are updated by the STDP rule [7, 8]. Here we use a simplified STDP rule [30]:

$$\begin{array}{c} \Delta w_{ij}=\{\begin{array}{cc} a^{+}w_{ij}\left(1-w_{ij}\right), & \;\text{if}\;t_{j}<t_{i},\\ -a^{-}w_{ij}\left(1-w_{ij}\right), & \;\text{elsewise}, \end{array} \end{array}$$ (2)

where *w*_*ij*_ is the weight of the synapse from the *j*th neuron in input layer to the *i*th neuron in convolutional layer, *t*_*i*_ and *t*_*j*_ are the corresponding firing time of two neurons, and *a*^+^ and *a*^−^ are the learning rates of STDP. This simplified rule ignores the exact spike time difference, because the input layer encodes the input signal into spikes within a short time duration. Note that even if no presynaptic spike occurs, the weight is still decreased. The term *w*_*ij*_(1 − *w*_*ij*_) as a soft bound limits the weight value between 0 and 1. The learning process will be ceased when the change of weight values is so small enough (|Δ*w*| < 0.01) that they have no effect on the final network performance on the test dataset.

To make the feature maps more distinct and their responses sparser, we further use a mechanism of lateral inhibition, which plays an important role in the auditory cortex [49]. With this mechanism, after a neuron fires, all the neurons in the same position in other feature maps are inhibited thereafter until the next sample appears, thus there is at most one spike allowed in each position during the processing of a sample. And after STDP is triggered on a neuron, the neurons in its neighborhood and all neurons at the same position in other feature maps are not allowed to perform STDP until next sample appears. With this competition mechanism, the feature maps are encouraged to learn different features.

### Pooling layer

The pooling layer performs a pooling operation on the convolutional layer to reduce the dimension of the representation, see Fig 1. Each feature map in the convolutional layer is processed independently, so the number of feature maps in the pooling layer is the same as that in the convolutional layer.

Each neuron in the pooling layer integrates inputs from one section in the corresponding feature map in the convolutional layer. The pooling neurons are not allowed to fire and their final membrane potentials are used as training data for a linear classifier. It should be noted that the pooling layer is not trained, and the value of its weights are fixed to 1. So the final potential of a pooling neuron can be seen as the spike number in its corresponding section and feature map in the convolutional layer. After the processing of each sample, the membrane potentials of pooling neurons are also reset.

### Evaluation

**Fig 3:**
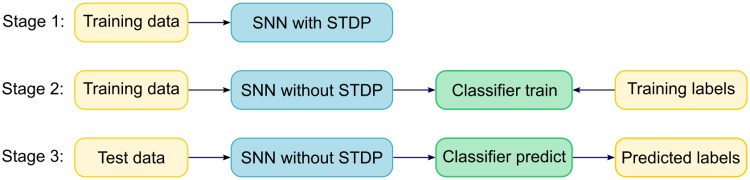
Three stages of model’s evaluation. Firstly, the SNN is trained with STDP on the training set without supervisory labels. Then the fixed network is run on the training set, and the output of the pooling layer and the corresponding labels in the training set, i.e. training labels, which are the corresponding labels of currently processed input samples, are used to train the classifier. Finally, the classifier is run to predict the labels of the test data, which are called as predicted labels. The classification accuracy of the model is evaluated by comparing the predicted labels with the corresponding ground truth labels.

We evaluate the model with a linear classifier. In detail, the evaluation of the model includes three stages, see Fig 3. First the SNN model is trained on the training set with STDP adjusting its synaptic weights. After the training is complete, we fix the weights by turning off the plasticity, run the network on training set and train the linear classifier on the membrane potentials of the pooling layer and the corresponding labels. Finally, we run the fixed network on the test set, and use the trained classifier to predict the labels to get the classification accuracy.

## Results

Our model was evaluated on the task of speaker-independent recognition of isolated spoken words with the TIDIGITS dataset [33] and the TIMIT dataset [35]. In this section, first we show the performance of our SNN model by using SVM as a classifier, which is compared with performances of other SNN and ANN models. Next, we validate the advantage of the local weight sharing strategy. Then, we analyze the transformation of receptive fields of the convolutional neurons and the characteristics of the network output to understand why our model works so well. Finally, we prove that our SNN model can also work well with a spike-based classifier by taking tempotron as an example.

### Performance on TIDIGITS dataset

We used utterances of female and male adults from the TIDIGITS dataset, which includes more than 4000 samples from 200 speakers. The dataset was randomly ordered and split into training set and test set with the ratio 7:3. In the experiment, there were 40 neurons in the input layer. The convolutional layer consisted of 50 feature maps, and the IF neurons in which had thresholds of 23. The convolutional window size was 6 × 40, which made it span all frequency bands, and its stride was 1. We divided the convolutional layer into 9 non-overlapping sections to share weights locally, and each section had a length of 4. The weights of convolutional layer were initialized with random values sampled from a Gaussian distribution with mean of 0.8 and standard deviation of 0.05. The learning rates of the STDP rule were *a*^+^ = 0.004 and *a*^−^ = 0.003.

**Fig 4:**
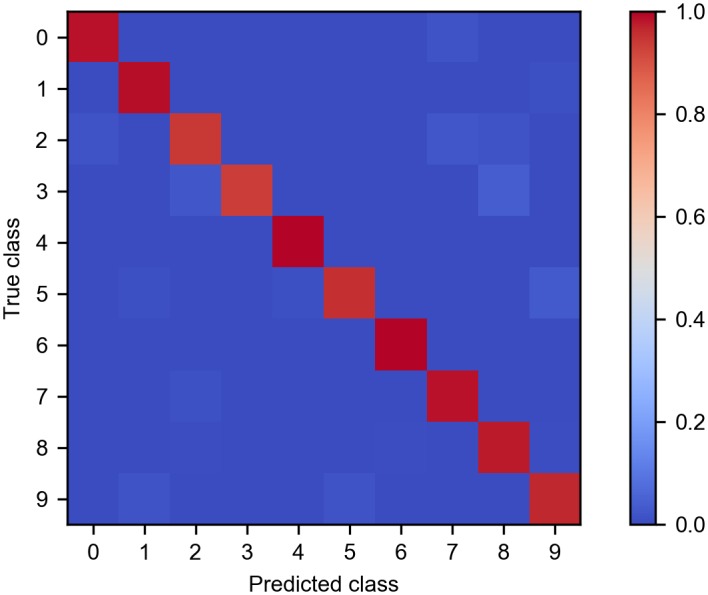
Confusion matrix of the classification result on the test set. The diagonal values indicate the ratio of correct classifications for each digit, while off-diagonal values represent the misclassifications.

After the SNN was trained by STDP, the output of pooling layer was classified using a linear SVM. With the parameters listed above, the model was able to achieve a classification accuracy of 97.5% on the test dataset. For a more detailed analysis, Fig 4 shows the confusion matrix of the test result which reveals the recognition rate for each spoken digit checked against all other digits and itself. Each row of the matrix represents an actual digit class while each column corresponds to a predicted digit class. According to Fig 4, recognition accuracy on digit 4 and digit 6 was 100%. The comparison of performance with other SNN models for spoken digit recognition is shown in Table 1. Among the SNN models, our model has the highest performance, which is also as good as the best result of ANNs. It should be noted that the comparison is not precise, because these studies either used different datasets, or used different ways of subsetting and splitting the dataset. For example, although some SNN models [19, 29] achieved high accuracies, they used smaller and easier datasets.

**Table 1:** Comparison of proposed SNN and other models on the isolated spoken digit classification task. RC: Reservoir computing; FC: Fully-connected layer; CSNN: Convolutional spiking neural network; HTM: Hierarchical temporal memory; RNN: Recurrent neural network; DN: Delta network.

Model	Architecture	Learning method	Dataset	Speakers	Accuracy
SNNs					
Verstraeten et al. [19]	RC	Pseudo matrix inversion	TI46	5	>97.5
Zhang et al. [21]	RC	Abstract learning rule	TI46	16	92.3
Wade et al. [27]	FC	STDP/BCM	TI46	16	95.25
Tavanaei et al. [28]	FC	Hebbian/anti-Hebbian STDP	Aurora	50	91
Tavanaei et al. [29]	CSNN	STDP	Aurora	>50	96
Dibazar et al. [50]	FC	Backpropagation	TIDIGITS	<80	85.1
**Our model**	CSNN	STDP	TIDIGITS	200	97.5
ANNs					
van Doremalen et al. [51]	HTM	Coincidence memorization	TIDIGITS	150	91.43
Neil et al. [52]	RNN	Backpropagation	TIDIGITS	200	96.1
Neil et al. [53]	DN	Backpropagation	TIDIGITS	200	97.5

**Fig 5:**
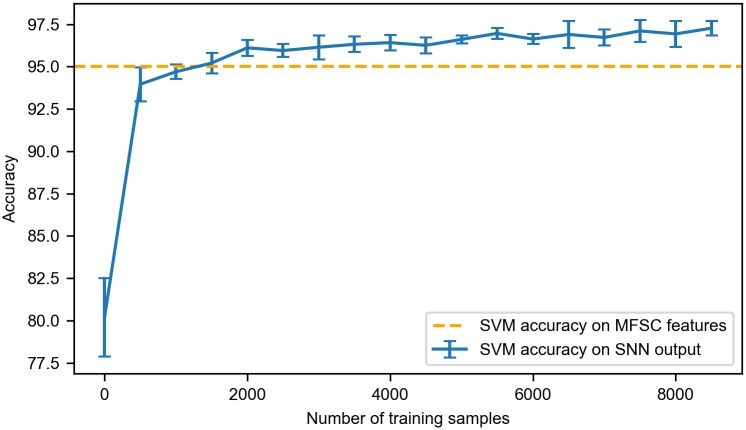
The SVM classification accuracy curve on the test set averaged over five runs. The SVM accuracy on the SNN output exceeded the accuracy on the MFSC features after 900 samples. The accuracy converges to about 97.5% after 6000 samples.

We evaluated the performance of the model at different time points through the training process, as shown in Fig 5. As the training proceeds, the test accuracy quickly increases along with the emergence of learned features. With merely 900 samples, the SVM accuracy on the SNN output exceeded the SVM accuracy of 95% on the MFSC features. After trained by approximately 6000 training samples, the model’s performance converges to about 97.5% and stays stable thereafter. The similar convergence behavior could also be found with a range of other parameter sets.

### Effect of local weight sharing

**Fig 6:**
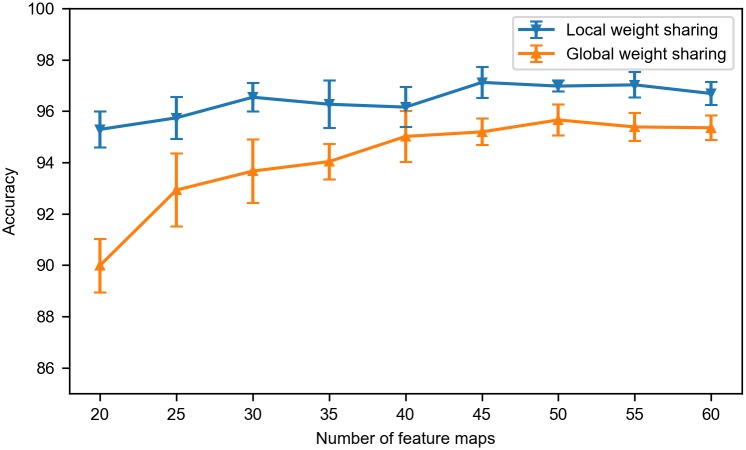
Performance comparison of local weight sharing and global weight sharing averaged over five runs. Global weight sharing works well with a larger number of feature maps, while local weight sharing performs significantly better than global weight sharing with fewer feature maps.

To show the advantage of the local weight sharing over the global weight sharing, we compared the performances of the SNN model with both strategies. With global weight sharing, all convolutional neurons in a feature map share their input synaptic weights, which can be viewed as that the convolutional layer only has one section. While in the case of local weight sharing, the convolutional layer has multiple sections which all have their own set of shared weights. The performance comparison between SNNs with these two mechanisms is shown in Fig 6. Each strategy was evaluated with different number of feature maps. With all different numbers of feature maps each strategy was evaluated, the local weight sharing outperformed the global weight sharing. Furthermore, as the number of feature maps decreased, the performance of global weight sharing droped quickly, while the SNN with local weight sharing could still maintain a high performance, since SNN with local weight sharing is better suited to learn local features of the speech signal. Therefore, the local weight sharing is more efficient than the global weight sharing.

### Evolving receptive fields

**Fig 7:**
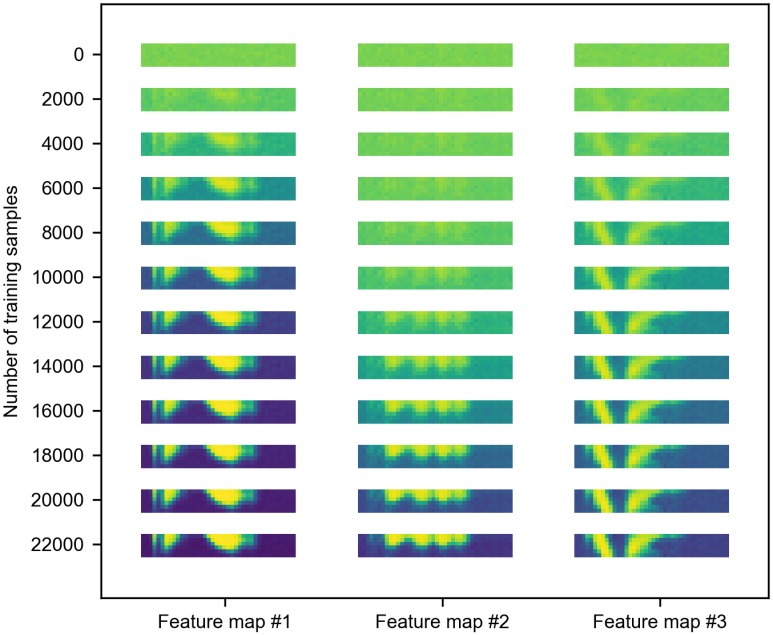
The visualization of evolving receptive fields in the training process. There is no special feature before training since the weights are initialized randomly. As the training proceeded, the features are gradually learned. Finally, the learned features are distinct from each other due to the effect of lateral inhibition.

To understand the forming process of receptive fields of the convolutional neurons, and provide insight into the dynamic learning behavior, we visualized the weights of the convolutional layer during the learning process. For this purpose, the weights of the convolutional layer were arranged into the convolutional window shape 6 × 40, and the weight values were used as the values of the corresponding pixels. Three randomly selected feature maps in the fifth section were visualized as examples in Fig 7 to show their evolving receptive fields through the training process. Before the training started, the weights were initialized with a Gaussian distribution, so the images in first column act as pure noise. During the training, the neurons which had more similar patterns with the input would reach the firing threshold earlier, and trigger the STDP rule to update its input synaptic weights and prevent other neurons from firing via the lateral inhibition mechanism. As the training of SNN proceeded, patterns arose as these feature maps started to learn features from the input, and the neuronal activations in the convolutional layer began to reflect the presence of their corresponding features in the original speech signal. Compared to the spectrogram of audio in Fig 2, the learned features have smoother edges, because with enough training data, STDP tends to learn more salient features and ignores the noise. Due to the lateral inhibition mechanism, all learned features are distinct from each other.

### SNN as an effective and efficient encoder

**Fig 8:**
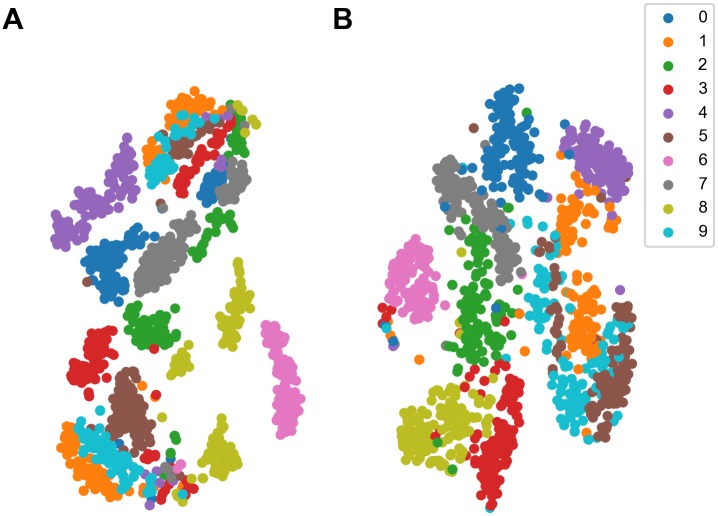
Visualization of MFSC features and SNN output with t-SNE. All samples are color-coded according to their digit classes. (A) The t-SNE visualization of MFSC features. Most digit classes have more than one cluster. (B) The t-SNE visualization of SNN output. The processing of SNN makes the clusters of each digit merged or closer.

In the experiment, the classification on the MFSC features with SVM achieved an accuracy of 95%, which is worse than the classification on the output of our SNN. This difference of results can be intuitively observed with the help of t-SNE technique [54], which is a powerful tool for visualizing high dimensional data. Fig 8 shows the t-SNE visualization of the difference of data separability between the MFSC features and the SNN output after training. In Fig 8A, the digits 0, 2, 3, 7, and 8 are grouped into several clusters respectively, but after the processing of SNN, they all have one cluster as shown in Fig 8B. Although the clusters of digit 1, 5, and 9 are not merged into one cluster for each digit, they become closer. The rest of digits 4 and 6 all have one cluster both before and after the processing. Therefore, after the encoding of the SNN, the data points of same digit become closer, and the digits become more separable, so that classifiers can have better performance in the output space than in the original space.

Although the increase of data separability is also a feature of reservoir computing, they usually achieve this target by increasing the dimension of data. In contrast, our SNN maps the data into a lower dimensional space. With 9 sections in the convolutional layer and 50 feature maps, the dimension of the SNN output is 450, while the MFSC features have a dimension of 1640, so our SNN reduces the data dimension by more than 70%. The reduction of dimension helps to remove redundant information and reduces the required computing time and storage space. For example, by running on a workstation with the Intel Core i7-6900K processor (3.2 GHz), the training of SVM takes 70 seconds on the MFSC features, but only takes 2 seconds on the output of our SNN.

**Fig 9:**
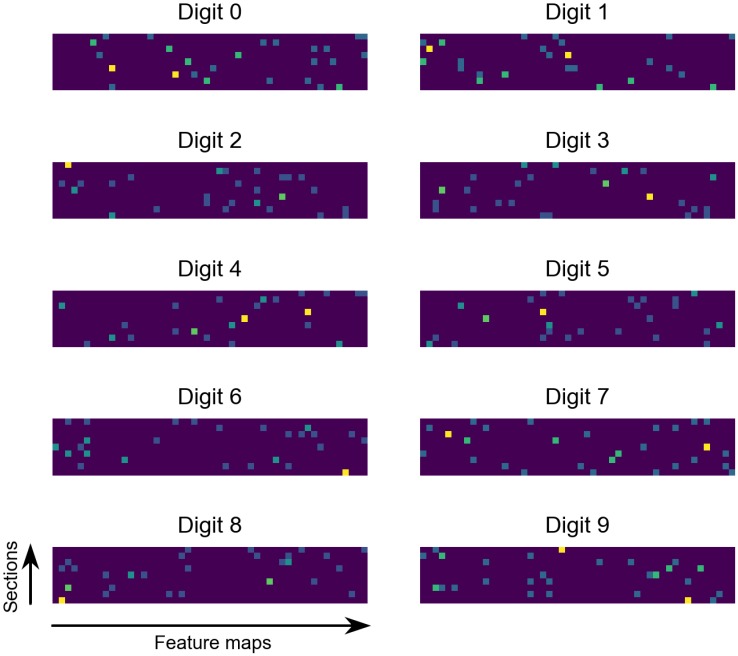
Visualization of SNN output. Each image represents the SNN output after a sample from the corresponding digit class is processed. The brightness of pixels represent the final membrane potentials of pooling neurons. Most pixels are dark in all images so the output is sparsely coded.

Fig 9 shows the visualization of pooling neurons’ activations for a random sample from each digit class, in which the final membrane potentials of pooling neurons are converted to the brightness of pixels. We can see from the figure that the output has a sparse representation. For each sample, only less than ten percent pooling neurons are activated, which acts as an efficient encoding of original data.

### Performance on a more difficult task based on the TIMIT dataset

**Fig 10:**
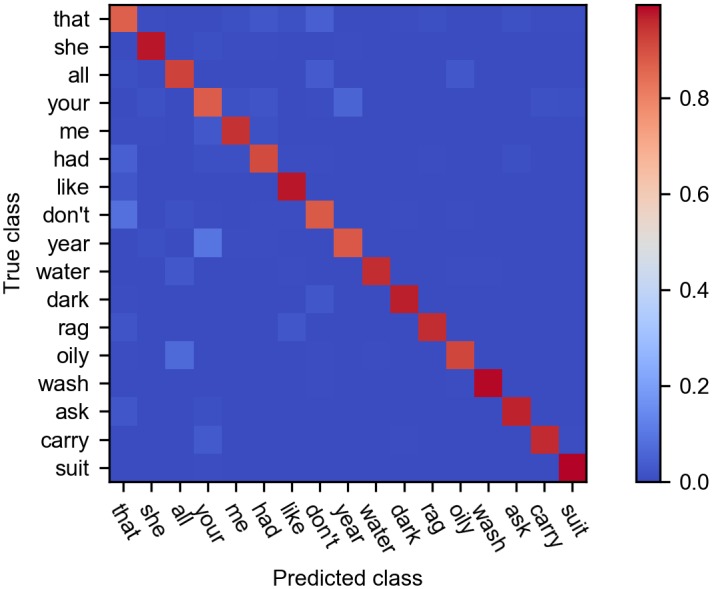
Confusion matrix of the classification result on the test set of the TIMIT dataset. The diagonal values represent the ratio of correct classifications for each word, and the off-diagonal values represent the misclassifications.

To show the capability of the proposed method on a more difficult SR task, we also tested the model on the TIMIT dataset, which contains 630 speakers of eight major dialects of American English. From this dataset we selected 17 words (shown in Fig 10) which are most frequent and not too short in length, and split these samples with the 7:3 ratio. In the task, the total number of feature maps in the convolutional layer was set to 70, and other parameters were the same as before. Although there are more categories (17) in this task comparing to 10 categories in the TIDIGITS dataset, the classifying accuracy by a linear SVM on the test dataset can be as high as 93.8%, which is just a little lower than that on the TIDIGITS dataset. Fig 10 shows the confusion matrix of the result on the test dataset.

### Comparison of different classifiers

To demonstrate the SVM classifier is not the necessary for our spiking neural network model, we also test the more biologically plausible classifiers, here we used a popular spike-based linear classifier—tempotron—as an example. Tempotron is a biologically plausible supervised learning model, which can discriminate different classes of inputs based on their spatiotemporal structures rather than their mean firing rates [34]. In the tempotron used here, the output of the pooling layer are converted to the spiking times of the input neurons in the tempotron model, where each classification neuron represents a category of inputs. During the training phase, if a classification neuron should fire for an input according to the corresponding label, while its maximal potential value fails to exceed its threshold, then the neuron’s synaptic weights are increased. And if a classification neuron should not fire, but its maximal potential value exceeds its threshold, its synaptic weights are then decreased. With the tempotron classifier substituting for the SVM, the classifying accuracies on the TIDIGITS dataset and the TIMIT dataset were 96% and 92.8%, respectively. Those results reveal that our SNN model could work well for SR tasks by using different kinds of classifiers.

## Discussion

Spiking neural networks had been gradually drawing attention due to its potential of solving ANNs’ problems of biological implausibility and computational intensity. However, it is not easy to train a SNN well for typical pattern recognition tasks, and various training methods have been proposed previously [13]. Many studies chose to train a traditional ANN instead, and convert it to a SNN by replacing each rate-based neuron with a spiking neuron [15, 16, 55–58]. Although they showed good performance on pattern recognition tasks, the problem of training a SNN was actually bypassed. Some researchers used differentiable formulations of SNNs, so they could train them with backpropagation directly [14, 59]. With this approach, the training algorithm searches a larger solution space and can achieve better performance. These methods are not biologically plausible since there are no evidence that error backpropagation could happen in the brain. In contrast, our model uses the STDP rule observed in biological synapses to train the SNN. Particularly, since STDP is a local and unsupervised learning rule, the training process doesn’t need any label information. Thus our SNN model is able to utilize the large amount of unlabeled data, which is less expensive and easier to obtain than labeled data. Moreover, a simple linear classifier (linear SVM or spike-based tempotron) can be sufficient to classify the STDP-trained data with high accuracies, this reveals powerful ability of our model for extracting input features in a more biologically realistic way.

There were other studies which also use STDP as the learning rule [28–31, 40]. Masquelier et al. [30] proposed a SNN which has a similar convolutional architecture to ours. The model has a four-layer hierarchy (S1–C1–S2–C2) where simple cells (S) gain selectivity from a linear sum operation, and complex cells (C) gain invariance from a max pooling operation. The S1 layer uses fixed Gabor filters to detect edges on various scaled versions of the input image. The S2 layer is selective to intermediate-complexity visual features. The C1–S2 synaptic connections are adjusted by STDP, and there is local inhibition between different S2 cells. The main difference between their network and ours is that the weights in their S2 layer are shared globally, while we use local shared weights to extract spatiotemporal features of acoustic signals, and it is more suitable for speech recognition tasks. Besides, we didn’t use various scaled versions of the input like they did, and our network completes the recognition task with only two layers, while they used one more layer with fixed hand-crafted weights. The work of Kheradpisheh et al. [31] was also inspired by Masquelier et al. [30], their model consists of multiple convolutional layers and pooling layers, this could benefit the image recognition task, in which all visual features can appear at any location of the input image. Compared to their work, our SNN only uses one convolutional layer and one pooling layer, as well as adopting local weight sharing instead of global weight sharing. Tavanaei et al. [29] also proposed a convolutional SNN for SR. In their model, the speech signal is converted into spikes trains using a Poisson process, from which the convolutional layer extracts primary acoustic features with shifted DoG filters, the generated feature maps are then pooled and sent to the feature discovery layer, which undergoes learning via a probabilistic STDP rule. The output of the network are used to train a hidden Markov model for evaluation. Our model is different from theirs in two ways. First, we use a more efficient temporal coding scheme instead of the rate-based Poisson process. Second, our model extracts primary acoustic features with STDP-trained receptive fields, while they extracted primary acoustic features by using shifted DoG filters, which are normally adopted to extract visual contrast informations, but there is no evidence of this mechanism in the auditory system.

Our model and the studies mentioned above have proven the effectiveness of STDP learning rule, but why STDP can work so well, since the construction of STDP-trained SNN models is often more heuristic than analytic? Actually, the learning process with STDP and lateral inhibition can roughly be seen as the sequential k-means (online k-means) algorithm [60]. In the sequential k-means algorithm, firstly initial centroids of data are guessed, then the data points are processed in a sequential order, each new data point is assigned to the closest centroid, and the corresponding centroid is moved closer to the data point after the assignment, these two steps are repeated until all data points are processed. With STDP and lateral inhibition, the learning process of the SNN is similar: the samples are fed into the SNN sequentially, for each input the neuron with the most similar receptive field will respond most strongly, and its weights will be updated by STDP to be more similar to its input, while the rest of neurons are laterally inhibited. For the convolutional structure in our model and the model in [30], the convolutional k-means method in [61] is comparable. Therefore, the convergence of k-means implies the convergence of STDP-based training. However, k-means algorithm may converge to a local minimum, so the STDP-based learning is likely to suffer from the same weakness.

One of the most important features of our SNN is the time-to-first-spike coding, which is faster and more efficient than the traditional rate coding. However, this coding scheme is ideal and highly simplified, since it assumes that all information about the stimulus is contained in the time of the first spike of a neuron, and all the following spikes are neglected. Rolls et al. [62] compared the information of the presented stimulus in the first spike and in the number of spikes in a given time window, and found that more information is available if all the spikes in the given time window are taken into account. Another weakness of the time-to-first-spike coding scheme is its vulnerability to noise, which means that even a single noise spike could heavily disrupt the information to be transmitted. On the other hand, the rate coding is inefficient but highly robust against noise, which is an essential feature of auditory system. Therefore, in the future research, the temporal coding scheme of utilizing all spikes responded to a stimuli should be considered to improve robustness as well as performance.

With regard to the application in industry, our model has the potential to be implemented on neuromorphic chips like TrueNorth [11] or Loihi, which can offer ultra-low power consumption. Although theoretically all SNNs can run on the neuromorphic chips during their prediction stage to save energy, for those SNNs which are trained by backpropagation directly or indirectly, the learning process still consumes significant power and time. However, with some chips support STDP learning rule [63–66], our SNN, as well as other SNNs trained by STDP, are able to achieve a low energy consumption even in the training stage.

Previous experimental studies had shown that there are both feedforward and feedback circuits in the auditory pathway [67], while our model only takes feedforward connections into consideration. Therefore, in the future, we could add feedback connections between layers into the network. With a recurrent structure, the signal related to supervised information can be sent back to each neuron through spikes for a more precise weight adjustment, thus a better performance may be achieved. It also should be noted that this is much different from that in typical reservoir computing models where recurrent but fixed connections are adopted.

## Conclusion

To provide an alternative speech recognition solution to ANNs which is biologically implausible and energy-intense, we proposed an STDP-based SNN model with the time-to-first-spike coding scheme and the local weight sharing strategy. It can achieve high accuracies on two speech recognition tasks. By adopting STDP learning rule and the temporal coding scheme, our SNN is able to learn acoustic features fast and efficiently, and make the speech data low-dimensional, sparse, and more linearly separable. Compared to global weight sharing, the proposed local weight sharing is more suitable for learning the features of speech signals. Moreover, our model can achieve comparable performance to traditional ANN approaches when using SVM as a classifier, and can also work well when using the spike-based classifier—tempotron. Therefore, in practice, due to the spike-based computation, our model with tempotron can be implemented on neuromorphic chips easily as a speech recognition solution with ultra-low power consumption. In summary, our study shows that a biologically plausible SNN model equipped with STDP, local weight sharing, and temporal coding has the ability of solving speech recognition tasks efficiently.
